# Anti‐Infective Effect of *Anacardium occidentale* L. Flowers Against Eskape Group Bacteria In Vitro, In Vivo, and In Silico

**DOI:** 10.1155/bmri/6579435

**Published:** 2026-07-20

**Authors:** Pâmela Gomes Santos, Josivan Regis Farias, Simone Batista Muniz, Danielle Cristine Gomes Franco, Ariadina Jansen Campos Fontes, Liane Maria Rodrigues Santos, Flavia Raquel Fernandes Nascimento, Claudia Quintino Rocha, Alexandra Martins dos Santos Soares, Rosane Nassar Meireles Guerra

**Affiliations:** ^1^ Laboratory of Immunophysiology, Universidade Federal do Maranhão—UFMA, São Luís, Maranhão, Brazil; ^2^ Graduate Program in Health Sciences, Center for Biological and Health Sciences, Universidade Federal do Maranhão—UFMA, São Luís, Maranhão, Brazil; ^3^ Graduate Program in Biotechnology—Center for Biological and Health Sciences, Universidade Federal do Maranhão—UFMA, São Luís, Maranhão, Brazil; ^4^ Laboratory of Chemistry and Natural Products—Center for Exact and Technological Sciences, Universidade Federal do Maranhão, São Luís, Maranhão, Brazil, ufma.br

**Keywords:** adhesion, biofilm, ciprofloxacin, sortase A, *Staphylococcus aureus*, *Tenebrio molitor*

## Abstract

**Background/Objectives:**

The study evaluated the in vitro antibacterial activity of the ethanolic extract of *Anacardium occidentale* flowers (EHAo) against ESKAPE pathogens and investigated the in vivo effects of the extract in *Tenebrio molitor* lethally infected with *Staphylococcus aureus*. In silico analyses investigated the affinity of compounds with sortase A.

**Methods:**

Chemical composition was characterized by HPLC and LC‐MS. Antibacterial activity was determined by minimum inhibitory concentration (MIC), minimum bactericidal concentration (MBC), and assays of adhesion and biofilm formation using *S. aureus*, *Enterococcus faecalis*, *Escherichia coli*, *Klebsiella pneumoniae*, and clinical MRSA strains. In vivo toxicity and antimicrobial efficacy were assessed in T. molitor larvae infected with *S. aureus* (10^5^ CFU/mL) and treated with PBS, ciprofloxacin (1.56 *μ*g/mL), or EHAo (3.12–12.5 mg/mL). Molecular docking evaluated the interactions between the chemical compounds identified in EHAo and the sortase A active site.

**Results:**

EHAo contained shikimic acid, gallic acid, quercetin, isoquercetin, galloyl‐glucose, digalloyl‐glucose, and tetragalloylglucose isomers. The extract exhibited bactericidal activity (MIC 1.25–6.25 mg/mL), with the strongest effects against *S. aureus*, MRSA, and E. faecalis. EHAo inhibited adhesion and biofilm formation, except in *K. pneumoniae*. The extract showed low toxicity in vivo and was efficient in improving the larvae′s survival following lethal infection with *S. aureus*. All compounds showed affinity to sortase A active site. However, digalloyl‐glucose displayed the highest predicted affinity.

**Conclusions:**

EHAo reduces bacterial virulence and improves survival in *S. aureus*–infected larvae, an activity probably related to the bactericidal activity, inhibition of adhesion and biofilm formation, and interaction with the sortase A active site.

## 1. Introduction

Bacterial infections caused by multidrug‐resistant microorganisms, especially those belonging to the ESKAPE (*Enterococcus faecium*, *Staphylococcus aureus, Klebsiella pneumoniae, Acinetobacter baumannii*, *Pseudomonas aeruginosa,* and *Enterobacter* spp.) group, pose a major global health problem due to high morbidity and mortality. These pathogenic microorganisms show exceptional adaptability and elevated virulence [1–3]. Their resistance and high pathogenicity make infections challenging, instigating the demand for new antibacterial agents [4, 5]. In addition, their resistance to multiple antibiotic classes, including cephalosporins, fluoroquinolones, and carbapenems, increases the complexity of clinical management, particularly in critically ill patients such as those in intensive care units [4–8].

In the last two decades, humanity has faced four epidemics. COVID‐19 was the most recent and the fifth deadliest in human history. Critically ill patients infected with SARS‐CoV‐2 exhibited high rates of morbidity and mortality. Over 50% of COVID‐19 patient deaths were due to untreatable secondary infections caused by bacteria, many of which belong to the ESKAPE group, such as *Staphylococcus aureus*, considered an opportunistic microorganism associated with these infections [3, 9].

The virulence of *S. aureus* infections is frequently related to the presence of enzymes such as sortase A (SrtA), a cysteine protease that anchors surface proteins to the bacterial cell wall in gram‐positive bacteria by recognizing the LPxTG motif. This process is essential for bacterial adhesion, colonization, and biofilm formation, and to enhance and maintain the *S. aureus* virulence. For this reason, SrtA is a promising target to control the virulence factor, as its inhibition can disrupt host–pathogen interactions without directly harming bacteria [10].

Bacterial resistance is a global health problem due to several factors, including the low efficacy of available therapies and the increased morbidity and mortality associated with it. This crisis is exacerbated by the slow discovery of new antibiotics, limited innovation, and regulatory challenges. Therefore, there is growing interest in alternative medical strategies, particularly natural products and other nonconventional approaches, which deliver novel mechanisms of action, potential synergistic effects, and reduced likelihood of resistance emergence [11, 12]. As a result, plants emerge as promising sources for bioprospecting these compounds, and the World Health Organization (WHO) encourages the search for new antimicrobial bioactive compounds for drug development or use as adjuvants [13]. The emphasis on new bioactive sources underscores the growing prominence of natural products as alternatives for treating various infections. This is especially relevant in traditional communities, where plants are frequently the primary source of therapeutics.

*Anacardium occidentale,* a medicinal plant popularly known as cashew tree, is native to Brazil and widely distributed throughout the country. Although native to Brazil, this species is also grown in other countries of tropical America from Mexico to Peru, as well as in countries in Asia and Africa. This species is a large tree with tall, twisted trunks, and glabrous simple, alternately arranged leaves. Its fruit—a chestnut—is appreciated gastronomically and called cashew. The floral peduncle, popularly known as “caju” in Brazil, is used to prepare juices, jams, and jellies [14, 15]. Cashew flowers are small pentamers with five petals and sepals, varying in color from white to red, mostly pink, and are arranged in panicle inflorescences at branch ends. Each tree bears both male and hermaphroditic flowers; only the hermaphroditic flowers, once fertilized, produce fruit. Cashew tree flowering occurs from June to November [15, 16].

Ethnobotanical data show the use of *A. occidentale* for various therapeutic purposes, including the treatment of fungal and bacterial infections. Several key studies have demonstrated the antimicrobial potential of various parts of the cashew tree, including bark, leaves, fruit, and floral peduncles, against a range of bacterial and fungal pathogens [14–19]. For example, Silva et al. [14] reported significant antibacterial activity of *A. occidentale* leaf extracts against *S. aureus* and *Escherichia coli*, whereas other studies [15–17] highlighted the potential of bark and fruit extracts in inhibiting clinically relevant microorganisms. Despite this growing body of evidence, the biological potential of cashew flowers as antimicrobials is still underexplored, and the majority of results belong to our research group [14, 19].

Based on this, our guiding hypothesis is that the ethanolic extract of *Anacardium occidentale* flowers (EHAo) may act as an effective antibacterial and antivirulence agent against ESKAPE pathogens. Therefore, this study aimed to: [1] evaluate the antimicrobial activity of EHAo against ESKAPE bacteria, including the effects on adhesion and biofilm formation; (ii) investigate the *in vivo* efficacy of EHAo during the lethal infection of *S. aureus* in *Tenebrio molitor* larvae; and (iii) explore, by *in silico* analysis, the interaction of chemical compounds with SrtA. Our goal is to advance plant‐derived alternatives for the treatment of drug‐resistant infections.

## 2. Materials and Methods

### 2.1. The Hydro‐EHAo

The *A. occidentale* flowers collection occurred in October 2024, with a cutting instrument, in the morning at the Federal University of Maranhão, São Luís campus (geographic coordinates: 2°55 ^′^34 ^″^ S, 44°30 ^′^58 ^″^ W). The Herbarium of Maranhão identified the botanical material (Exsiccate Number 14.709). The study was registered at SisGen AAA8E4B.

For extraction, the flowers were dried at room temperature (25°C ± 3°C) for 7 days in the dark. The dried material was crushed in a knife mill, yielding a powder. This powder was used to obtain the ethanolic extract by exhaustive maceration in absolute ethanol P.A. The process was repeated every 7 days for 3 weeks. Throughout extraction, the solvent‐to‐biomass ratio was 5:1 to ensure precise reproducibility [14, 19].

Following the extraction, the EHAo was evaporated using a rotary evaporator and dried by cold jet, yielding 65.21 g with an average yield of 36.31%. In the final step, the extract was stored in sterile, dark flasks at 4°C until used in the experiments.

### 2.2. Chemical Evaluation by High‐Performance Liquid Chromatography (HPLC) Coupled to Mass Spectrometry—LC‐ESI‐IT/MS.

HPLC is an analytical technique used to separate, identify, and quantify compounds in a mixture. When coupled with mass spectrometry (LC‐ESI‐IT/MS), it allows for mass analysis of separated compounds for detailed chemical characterization. ESI stands for electrospray ionization, a technique that produces ions using an electric charge. This refers to the type of mass analyzer. Chemical characterization was performed by LC‐ESI‐IT‐MS with a spectrometer (Amazon SL Bruker Daltonics, Massachusetts, United States).

Chromatographic analysis was conducted on a Luna 5 *μ*m C18 100 Å column (250 × 4.6 mm, Phenomenex, Torrance, United States) with a Shimadzu Prominence HPLC. The mobile phase in the binary gradient consisted of formic acid (0.1%—Sigma‐Aldrich, St. Louis, Missouri, United States) in water (solvent A) and formic acid in methanol (0.1%—Sigma‐Aldrich, St. Louis, Missouri, United States) (solvent B). Samples (1 mg mL^−1^) were eluted from the column with a gradient from 5% to 100% solvent B. The flow rate was constant at 1 mL min^−1^ for 40 min. The injection volume was 2 *μ*L.

The speaker compartment temperature was set to 40°C. Data acquisition was performed in negative‐ion mode, with fragmentation at multiple stages (MS2 and MS3). The following parameters were used: nebulization gas pressure of 50.0 psi; capillary temperature of 300°C; transfer capillary input voltage of 4500 V; dissolving gas (nitrogen, N2) at a flow of 10 L min^−1^; collision gas (helium [He]); and acquisition range of m/z 50–1200. The raw data were analyzed using Data Analysis 4.3 software (Bruker, Massachusetts, United States).

### 2.3. Evaluation of the Antimicrobial Activity of EHAo In Vitro

#### 2.3.1. Microorganisms

Standard strains of gram‐positive bacteria included *Enterococcus faecalis* (ATCC 29212) and *S*. *aureus* (ATCC 25923). Gram‐negative strains included *E*. *coli* (ATCC 25922) and *Klebsiella pneumoniae (ATCC 700603).* All were cultured at the Immunophysiology Laboratory of the Federal University of Maranhão. Prof. Dr. Valerio Monteiro Neto at the Center for Basic and Applied Immunology provided the multidrug‐resistant clinical lineages methicillin‐resistant *Staphylococcus aureus* (MRSA) (240016969962) and MRSA (240016969961). For antimicrobial evaluation tests, the bacteria were reactivated in Mueller–Hinton (MH) broth at 37°C for 24 h.

#### 2.3.2. Determination of the Minimum Inhibitory Concentration (MIC)

The antimicrobial assay was performed by the microdilution method in sterile 96‐well flat‐bottom microplates, according to the M7‐A6 standard described by the Clinical and Laboratory Standards Institute [20]. The standard strains of *E. faecalis*, *S. aureus*, *E. coli,* and *K. pneumoniae,* and the clinical MRSA strains were reactivated and seeded in MH (Merck, Brazil) and incubated at 37°C for 24 h. The inoculum was adjusted to 0.5 on the McFarland scale (1.5 × 10^8^ CFU/mL). The suspension was homogenized (Vortex) for 15 s, and the cell density was adjusted using a spectrophotometer (600 nm). This produced a suspension corresponding to 0.5 of the McFarland scale; comparison with a standard solution gave about 1.5 × 10^8^ CFU/mL [21].

To determine the MIC, the bacterial cultures suspended in MH broth (10 *μ*L) were added to wells containing different concentrations of EHAo (200–0.391 mg/mL). Ciprofloxacin (CIPRO) (200–0.390 *μ*g/mL) served as the positive control. DMSO (Dimethylsulfoxide, Sigma‐Aldrich; 2% in saline), used to increase the extract′s solubility, served as the negative control. The plates were incubated for 24 h at 37°C, and the absorbance was measured (600 nm), followed by resazurin staining for viability confirmation and to determine the effects of EHAo on bacterial growth. All samples were tested in sextuplicate.

The MIC was the lowest concentration that stopped visible growth. The MBC/MIC ratio was used to determine if EHAo was bactericidal (MBC/MIC < 4) or bacteriostatic (MBC/MIC > 4) [22].

The MBC was determined from MIC results in Petri dishes containing Mi; 10 *μ*L from each MIC value was used. After incubation at 37°C for 24 h, the concentrations at which no bacterial growth occurred were classified as inhibitory. When observed, the formation of more than three colony forming units (CFU) indicated a noninhibitory concentration for growth [23].

#### 2.3.3. Adhesion and Eradication of Preformed and Formed Biofilms

Eradication of adhered, preformed, and formed biofilms of *S*. *aureus* (ATCC 25923), MRSA (240016969662), and *E. coli* (ATCC 25922) was evaluated as described previously [21]. Biofilm biomass was measured with crystal violet, and cell viability was checked using MTT (3‐4,5‐dimethyl‐2‐thiazolyl bromide)‐2,5‐diphenyl‐2H‐tetrazolium.

For the adhesion and preformed biofilm assay, first, MH broth was added to the wells of a flat‐bottom 96‐well culture plate. The bacterial isolates (10 *μ*L) were then introduced, followed by treatment with subinhibitory concentrations of 1/2 and 1/4 of the MIC of the extract and CIPRO, which was used as a positive control (CIPRO). Finally, the plates were incubated for 90 min and 24 h at 37°C, respectively.

To evaluate the effects of EHAo against the formed biofilm, MH and bacterial isolates were added to the plates. After incubation for 24 h, the plates were washed twice with PBS and treated with suprainhibitory concentrations of the extract or CIPRO, corresponding to 2x and 4x the MIC of the EHAo. Then, the plates were again incubated for 24 h at 37°C. After incubation, the contents of the wells were removed, and the remaining material was washed twice with PBS. Then, 100 *μ*L of MH was added to each well, followed by 50 *μ*L of MTT (3‐(4,5‐dimethylthiazol‐2‐yl)‐2,5‐diphenyltetrazolium bromide, Sigma‐Aldrich, United States), and the plates were incubated for 2 h at 37°C. Absorbance was measured at 570 and 600 nm.

The concentrations at which no bacterial growth occurred were classified as inhibitory. When observed the formation of more than three CFU, the concentration was noninhibitory to growth [23].

### 2.4. In Vivo Assays in *T. molitor* Larvae

#### 2.4.1. Acute Toxicity Assessment

The acute toxicity test was performed as described previously [24, 25] with adaptations. For the assay, the selection for each group (*n* = 10 larvae/group) included larvae measuring 1.5–2.0 cm and viability. The viability considered motility, effective response after touch and absence of melanization. Table 1 summarizes the description of the experimental groups. EHAo values are 1/2 MIC (3.125 mg/mL); MIC (6.25 mg/mL); 2x MIC (12.50 mg/mL); 4x MIC (25 mg/mL); 8x MIC (50 mg/mL) and 16x MIC (100 mg/mL).

**Table 1:** Groups to assess the toxicity of EHAo on *Tenebrio molitor* larvae.

Groups	Treatment
Sham	—
DMSO	DMSO 2%
PBS	PBS
EHAo1/2	½ MIC—3.125 mg/mL
EHAo1	MIC—6.250 mg/mL
EHAo2	2x MIC—12.500 mg/mL
EHAo4	4x MIC—25.000 mg/mL
EHAo8	8x MIC—50.000 mg/mL
EHAo16	16x MIC—100.000 mg/mL

The survival was determined daily (every 24 h) for 168 h. The death was defined as the absence of response to touch, lack of motility, and melanization [26].

#### 2.4.2. Standardization of Lethal Inoculum of *S. aureus*

For the infection, *S. aureus* cultured in MH broth (24 h) was diluted in concentrations ranging from 10^1^ to 10^7^ CFU/mL [27]. The larvae were then infected intracelomic (5 *μ*L) and monitored for survival for 3 days (*n* = 5 larvae/group). The tests were performed in triplicate considering as control group that received PBS (5 *μ*L).

#### 2.4.3. Lethal Infection

The effect of EHAo on lethal infection was determined in five groups as shown in Table 2, considering the MIC values:

**Table 2:** Groups to evaluate the EHAo effect against *S. aureus* lethal infection.

Groups	Treatment (5 *μ*L/ic)
Control	PBS
CIPRO	Ciprofloxacin (1.56 *μ*g/mL)
EHAo1/2	½ MIC (3.12 mg/mL)
EHAoMIC	MIC (6.25 mg/mL)
EHAo2	2x MIC (12.50 mg/mL)

For the infection and treatment, the larvae were paralyzed by cooling with ice and received 5 *μ*L of *S. aureus* inoculum (1.5 × 10^5^ CFU/mL) intracelomically (IC). Treatment with EHAo occurred soon after, using the same route, in the fourth metamer, in the ventral portion. At the end, the larvae received standardized food. The survival was monitored daily for 3 days.

#### 2.4.4. Quantification of CFU in *T. molitor* Larvae Lethally Infected by *S. aureus*

The quantification of bacterial load (CFU) in *T. molitor* larvae was carried out as previously described by Oliveira et al. [19] and McGonigle et al. [27], with adaptations. The larvae (*n* = 10 larvae/group) were infected with *S. aureus* (5 *μ*L/ic) at a concentration of 1.5 × 10^5^ CFU/mL and were euthanized within 6 h. The CFU count was carried out after euthanasia. For the evaluation, the larvae were ground in 5 mL of sterile PBS, and the resulting suspension was diluted (10^1^–10^4^). Then, 100 *μ*L of the suspension at the lower dilution (10^4^) was transferred to Petri dishes containing Mannitol Salt Agar medium (Kasvi, São Paulo, Brazil). Afterwards, the CFU was determined after the incubation for 24 h at 37°C.

### 2.5. Molecular Docking and Interaction Analysis of Selected Natural Compounds With SrtA

Molecular docking simulations were performed to evaluate the interaction between EHAo compounds and SrtA from *S. aureus*. The three‐dimensional structure of the enzyme was retrieved from the Protein Data Bank (PDB ID: 1T2P; resolution: 2.00 Å). The protein was prepared in Molegro Virtual Docker (MVD) [28]. Only chain A was used for docking, whereas all other chains, the cocrystallized peptide, and water molecules were excluded. Structure preparation was performed under default parameters, including standard structural corrections and hydrogen addition.

The three‐dimensional structures of the ligands were retrieved in SDF format from PubChem (https://pubchem.ncbi.nlm.nih.gov/). These included shikimic acid (CID: 49867941); galloyl glucose derivatives such as 3‐O‐galloyl‐glucose (CID: 101371854) and 3‐galloyl‐glucose (CID: 118430668); gallic acid (CID: 370); digalloyl‐glucose derivatives, including 2,6‐digalloyl‐D‐glucose (CID: 54116668), 3,6‐digalloyl‐glucose (CID: 129633560), and 2,3‐digalloyl‐D‐glucose (CID: 129819858); as well as isoquercetin (CID: 10813969) and quercetin (CID: 5280343). Tetragalloylglucose was excluded from the docking analysis due to its structural features, which may compromise the reliability of molecular docking predictions.

The molecular structures were individually subjected to geometry optimization using the MMFF94 force field implemented in Avogadro [29]. Energy minimization was carried out employing the steepest descent algorithm under default convergence criteria. The optimization was repeated until energy convergence was achieved, as indicated by minimal variation between successive steps.

Docking simulations were performed using MVD, employing the MolDock Score [GRID] as the scoring function and MolDock SE as the search algorithm. All other parameters were kept at their default settings. The binding site considered the enzyme′s active site, as annotated in the UniProt entry Q9S446. The docking grid was centered at *x* = −39.93, *y* = −15.08, and *z* = 2.89, with a spherical search radius of 10 Å. Compounds were ranked according to the MolDock Score, with more negative values indicating higher predicted binding affinity. Visualization of the ligand–protein complex and interaction profile of the top‐ranked compound used its best docking pose in BIOVIA Discovery Studio Visualizer to identify key interacting residues.

### 2.6. Statistical Analysis

After tested to normality the results were evaluated by Student′s *t*‐test for two‐group comparisons or by analysis of variance (ANOVA) for several‐group comparisons, followed by the multiple‐comparison test (Newman–Keuls) [30], the Kaplan–Meier curve determined the survival, followed by the log‐rank test. The analyses used GraphPad Prism, Version 10.0. All data were graphed as mean ± standard deviation, with *p* ≤ 0.05 considered significant.

## 3. Results

### 3.1. Chemical Analysis and Identification of Substances Present in EHAo

**Figure 1:**
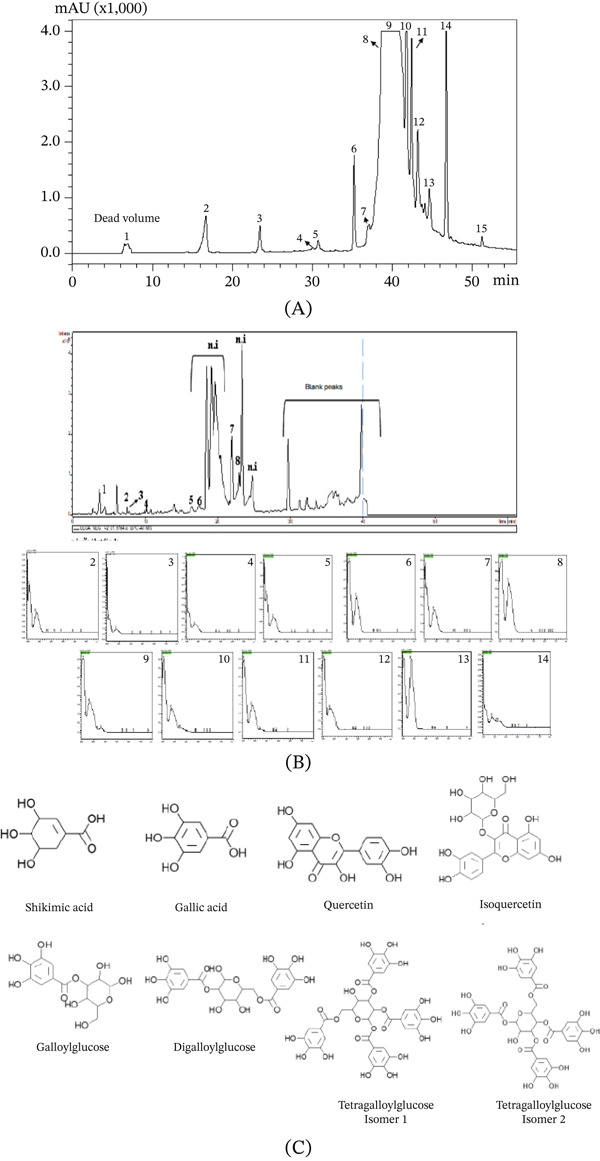
Chemical evaluation of the ethanolic extract of *Anacardium occidentale* L flowers: (A) UV‐Vis chromatogram at 254 nm, (B) total ion chromatogram (LC‐ESI‐IT/MS), and (C) identified compounds.

The extract showed retention times between 16 and 55 min, corresponding to 14 compounds, with different wavelength profiles, ranging from 270 to 365 nm (Figure 1A). The wavelengths in Figure 1A indicate the presence of acids from compounds present in the genus *Anacardium*. Using LC‐MS, the following compounds were identified in the extract: shikimic acid, gallic acid, quercetin, isoquercetin, galloyl glucose, digalloyl‐glucose, isomer 1 and isomer 2 of tetragalloylglucose (Figure 1B,C).

Table 3 shows the compounds identified, considered the number and wavelength profile after chemical characterization of the EHAo by HPLC‐ESI‐IT/MS.

**Table 3:** Compounds identified after chemical characterization of the ethanolic extract of *Anacardium occidentale* by HPLC‐ESI‐IT/MS.

Number	[M−H]^−^	MSN	Compound
**1**	173	155, 137, 111, 93	Shikimic acid
**2**	331	273, 169	Galloyl glucose
**3**	169	125	Gallic acid
**4**	483	405, 437, 383	Digalloyl‐glucose
**5**	787	617, 573, 467, 405	Tetragalloylglucose (Isomer 1)
**6**	787	617, 467, 291, 261	Tetragalloylglucose (Isomer 2)
**7**	483	303	Isoquercetin
**8**	301	—	Quercetin

### 3.2. Antimicrobial Activity of EHAo Against Bacteria of the ESKAPE Group

Table 4 shows that the MBC/MIC ratio of 2 for all tested bacterial samples indicates a bactericidal profile of the EHAo. The extract showed antibacterial activity *in vitro* against all microorganisms tested, with the best activity against *S. aureus,* with MIC and MBC values of 6.25 and 12.5 mg/mL, respectively. The extract also exhibited antimicrobial activity against *E. faecalis* and *E. coli*, with MIC and MBC values of 12.5 and 25 mg/mL, respectively. However, EHAo was not effective for *K. pneumoniae* (MIC = 50 mg/mL and MBC = 100 mg/mL). Regarding MRSA strains, the extract was equally effective against all samples tested, with MIC (12.5 mg/mL) and MBC (25 mg/mL) values. CIPRO treatment was effective for all samples.

**Table 4:** Minimum inhibitory concentration (MIC) and minimum bactericidal concentration (MBC) of the EHAo in cultures of gram‐positive bacteria; gram‐negative and clinical samples of bacteria from the ESKAPE group.

Bacteria (10^8^ CFU/mL)	EHAo^a^ (mg/mL)		MBC/MIC	Ciprofloxacin (*μ*g/mL)	
	MIC^b^	MBC^c^		MIC	MBC
*Staphylococcus aureus* (ATCC25923)	6.25^d^	12.50	2.0	1.56	3.12
MRSA	12.50	25.00	2.0	200.00	200.00
MRSA (240016969961)	12.50	25.00	2.0	12.50	12.50
MRSA (240016969962)	12.50	25.00	2.0	1.56	12.50
*Enterococcus faecalis* (ATCC29212)	12.50	25.00	2.0	2.60	5.20
*Escherichia coli* (ATCC25922)	12.50	25.00	2.0	3.12	6.25
*Klebsiella pneumoniae* (ATCC700603)	50.00	100.00	2.0	2.08	4.16

^a^EHAo: ethanolic extract of *A. occidentale* flowers.

^b^MIC: minimum inhibitory concentration.

^c^MBC: minimum bactericidal concentration.

^d^The values correspond to the average of triplicate.

### 3.3. EHAo Reduced the Adhesion and Biofilms of *S. aureus*, MRSA and *E. coli*

**Figure 2:**
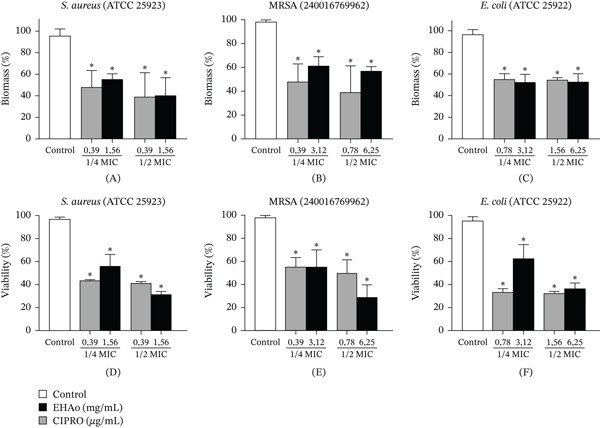
The ethanolic extract of *A. occidentale* flowers (EHAo) inhibited the adhesion of *S. aureus* (A, D), MRSA (B, E), and *E. coli* (C, F), as evidenced by reductions in biomass (A–C) and viability (D, F). Adhesion inhibition was determined in bacterial cultures treated with subinhibitory concentrations corresponding to 1/4 and 1/2 of the MIC values of the extract (EHAo) and ciprofloxacin (CIPRO) for each culture and compared with untreated cultures (Control). The evaluation considered the optical density (570 nm). The data correspond to the mean ± standard deviation of samples tested in quintuplicate. ( ^∗^) *p* ≤ 0.05 in relation to the untreated control and (^#^) in relation to the CIPRO group.

The extract, when used at subinhibitory concentrations (1/2 CIM and 1/4 MIC), was effective to inhibit adhesion in *S. aureus* (Figure 2A), MRSA (Figure 2C), and *E. coli* (Figure 2B) samples, as indicated by biomass. Similar results were observed regarding viability (Figure 2D–F). In addition, the highest concentration of EHAo reduced the adherence to values similar to that of antibiotics.

**Figure 3:**
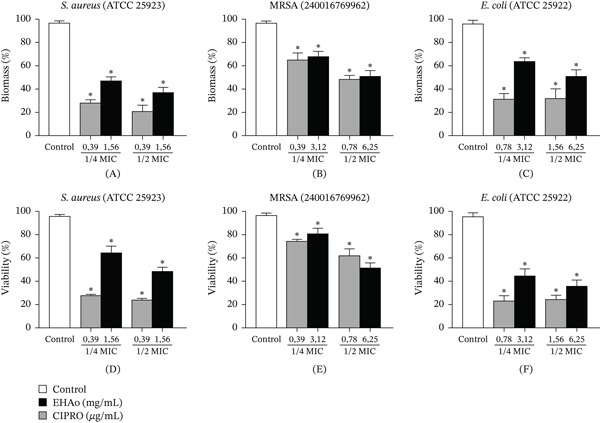
The ethanolic extract of *Anacardium occidentale* (EHAo) inhibited the evolution of the preformed biofilm of *S. aureus* (A, D), MRSA (B, E), and *E. coli* (C, F), considering the effects on biomass (A–C) and on the viability of bacterial cultures treated with subinhibitory concentrations corresponding to the EHAo (1/4 and 1/2 MIC) and ciprofloxacin (CIPRO). The results obtained for optical density (570 nm) correspond to the mean ± standard deviation of samples tested in quintuplicate. ( ^∗^) *p* ≤ 0.05 in relation to the control and (^#^) in relation to the CIPRO group.

The treatment with EHAo inhibited the preformed biofilms (Figure 3) of all bacterial cultures tested, when the biomass (Figure 3A–C) or viability (Figure 3D,F) was evaluated. Treatment with either CIPRO or EHAo was less effective for MRSA cultures (Figure 3B,E).

**Figure 4:**
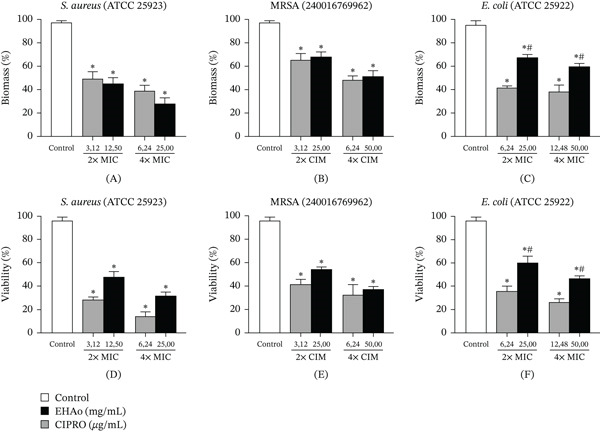
The ethanolic extract of *Anacardium occidentale* (EHAo) inhibited the biofilm formed from *S. aureus* (A, D), MRSA (B, E) and *E. coli* (C, F), considering the effects on biomass (A–C) and on feasibility (D–F) bacterial cultures treated with suprainhibitory concentrations of the extract (EHAo) and ciprofloxacin (CIPRO), corresponding to 2x and 4x MIC. The results obtained for optical density (570 nm) correspond to the mean ± standard deviation of samples tested in quintuplicate. ( ^∗^) *p* ≤ 0.05 in relation to the control and (^#^) in relation to the CIPRO group.

Treatment with inhibitory concentrations of the extract corresponding to 2x MIC and 4x MIC also inhibited the growth of the biofilm formed for all three cultures of bacteria tested, when compared with the untreated control, considering the effects on biomass (Figure 4A–C and viability (Figure 4D–F). The best results were for *S. aureus* (Figure 4A). (Figure 4B) and *E. coli* (Figure 4C). Treatment with EHAo inhibited the viability of biofilms formed in all samples tested, with greater efficacy against *S. aureus* (Figure 4D) and MRSA (Figure 4E), although it was also effective against *E. coli* (Figure 4F).

### 3.4. EHAo Showed Low Toxicity to *T. molitor Larvae*

**Figure 5:**
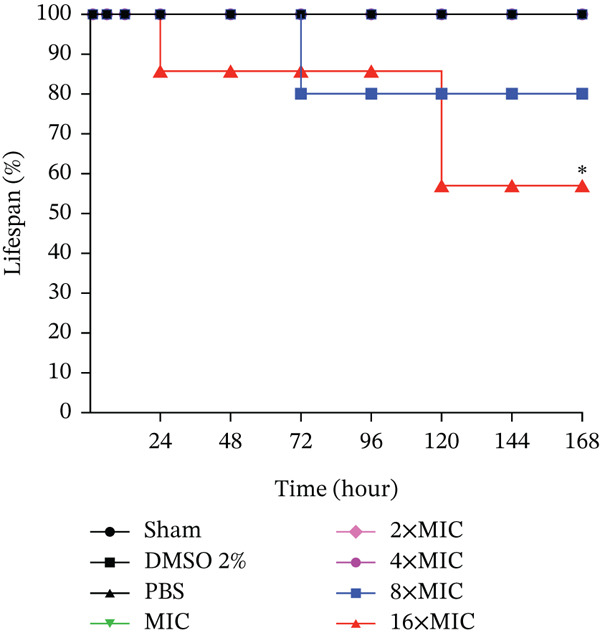
The ethanolic extract from *A. occidentale* (EHAo) flowers shows low toxicity to *Tenebrio molitor* larvae. The larvae received the treatment with EHAo by intracelomic injection at concentrations corresponding to MIC, 2x MIC, 4x MIC, 8x MIC, and 16x MIC. Monitoring was carried out every 24 h for 7 days (168 h). The data represent the mean ± standard deviation from three independent assays at each extract concentration, with 30 larvae/group. Mortality was assessed using Kaplan–Meier curves and the log‐rank test. ( ^∗^) *p* ≤ 0.05 in relation to the group treated with PBS.

The crude extract at MIC (6.25 mg/mL), 2x MIC (12.5 mg/mL), and 4x MIC (25 mg/mL) concentrations did not show toxicity to the larvae, as indicated by 100% survival at the end of the evaluation. However, there was a 20% reduction in survival at the concentration corresponding to 8x MIC (50 mg/mL) and 50% at the highest concentration (16x MIC, 100 mg/mL), indicating that this may be the IC50 for *T. molitor* larvae (Figure 5).

### 3.5. The Lethal Inoculum of *S. aureus* for *T. molitor* Larvae

**Figure 6:**
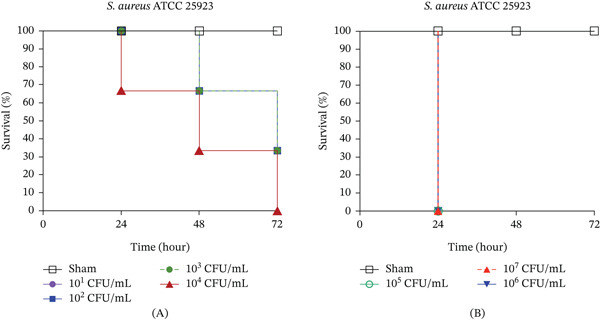
Determination of *S. aureus* inoculum for lethal infection in *Tenebrio molitor* larvae. Increasing bacterial concentrations (A) 101 to 104 CFU/μL and (B) 105 to 107 CFU/μL were used for infection. Monitoring was carried out for 3 days. The data represent the mean ± standard deviation from three independent trials, totaling 30 larvae/group. The analyses used Kaplan‐Meier curves, followed by the Log‐Rank statistical test. ( ^∗^) *p* ≤ 0.05 in comparison with untreated animals.

To evaluate the larvae′s susceptibility to S*. aureus* infection, the animals were infected with bacterial suspensions at concentrations ranging from 10^7^ to 10^1^ CFU/mL. The results show that inoculum between 10^5^ and 10^7^ CFU/mL (Figure 6A) produced similar results, with 100% of the larvae dying within 24 h after infection. Figure 6B shows that the group infected with 10^4^ CFU/mL, 100% died only after 72 h, that is, later. The smaller bacterial inoculum, ranging from 10^1^ to 10^3^ CFU/mL, showed low lethality, with 40% of the larvae surviving until the end of the evaluation (Figure 6B). Thus, since the results with the highest concentrations were similar, we chose the 10^5^ CFU/mL *S. aureus* inoculum for the other assays.

**Figure 7:**
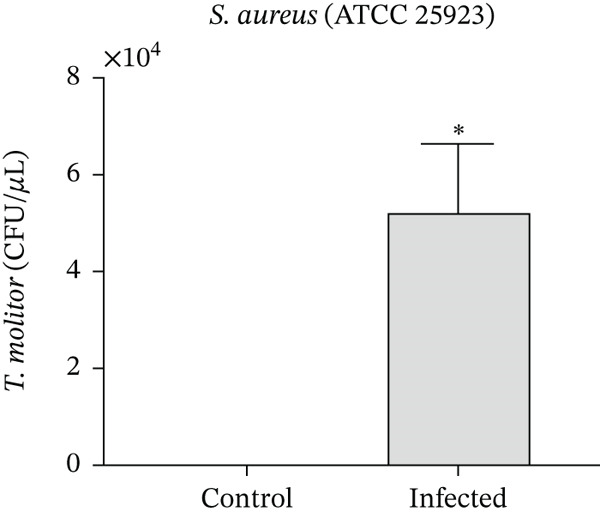
Number of Colony Forming Units (CFU) in (A) *T. molitor* larvae infected with *S. aureus* (ATCC25923). Groups with 10 larvae were infected with *S. aureus* (1.5 × 10^5^ CFU/mL) and euthanized after 6 h. The data were analyzed in relation to the non‐infected control, by Student′s test, of individual samples tested in triplicate with ( ^∗^) *p* ≤ 0.05.

Figure 7 shows an increase in the number of CFU *S. aureus* 6 h after infection in larvae of *T. molitor*. The bacterial load is much higher than in cultures of noninfected larvae, indicating successful infection induction.

### 3.6. Treatment With EHAo Prolongs the Survival of *T. molitor* Larvae After Lethal Infection With *S. aureus*

**Figure 8:**
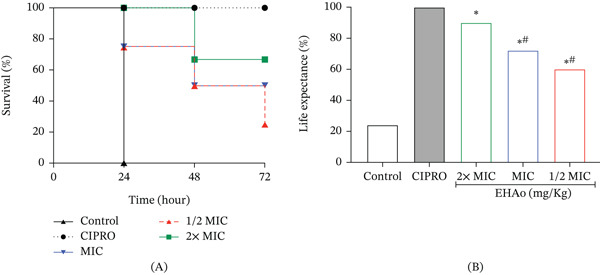
Treatment with the ethanolic extract of *A. occidentale* flowers (EHAo) increases (A) survival and (B) life expectancy of *T. molitor* larvae infected with *S. aureus* (ATCC 25923). Groups with 10 larvae were infected with *S. aureus* (1.5 × 10^5^ CFU/mL) and treated with different EHAo concentrations, intracelomically, in values corresponding to 2x MIC, MIC, and 1/2 MIC. Survival was followed for 72 h, considering *n* = 10 larvae/group. ( ^∗^) *p* ≤ 0.05 in relation to the infected and untreated control.

Figure 8(A) shows that the EHAo treatment ensured the survival of larvae lethally infected with *S. aureus* and that the 2x MIC concentration was the most effective, with 70% survival. In the EHAo‐treated groups at the MIC concentration, survival was 50%. Treatment with subinhibitory doses was not very effective in ensuring survival (20%).

Figure 8B shows the data on life expectancy. It is possible to observe a 90% life expectancy rate in the animals in group 2x MIC, 70% in the MIC group, and 60% in the 1/2 MIC group, still higher than that observed in the infected and untreated control.

### 3.7. In Silico Affinity EHAo Compounds With SrtA Active Site

Molecular docking analysis showed EHAo compounds displayed distinct binding affinities toward SrtA, as reflected by their MolDock scores (Table 5), in which more negative values correspond to higher predicted affinity. Among the tested molecules, digalloyl‐glucose and isoquercetin exhibited the most favorable scores, −104.02 and −99.07, respectively. In contrast, smaller molecules such as gallic acid and shikimic acid showed comparatively lower predicted affinities, −62.58 and −60.97, respectively.

**Table 5:** Molecular docking scores (MolDock Score) of EHAo compounds against *Staphylococcus aureus* sortase A.

Compound	MolDock Score
2,6‐digalloyl‐D‐glucose	−104.02
3,6‐digalloyl‐glucose	−99.07
Isoquercetin	−91.26
3‐O‐galloyl‐glucose	−83.90
3‐galloyl‐glucose	−82.68
2,3‐digalloyl‐D‐glucose	−78.91
Quercetin	−73.21
Gallic acid	−62.58
Shikimic acid	−60.97

**Figure 9:**
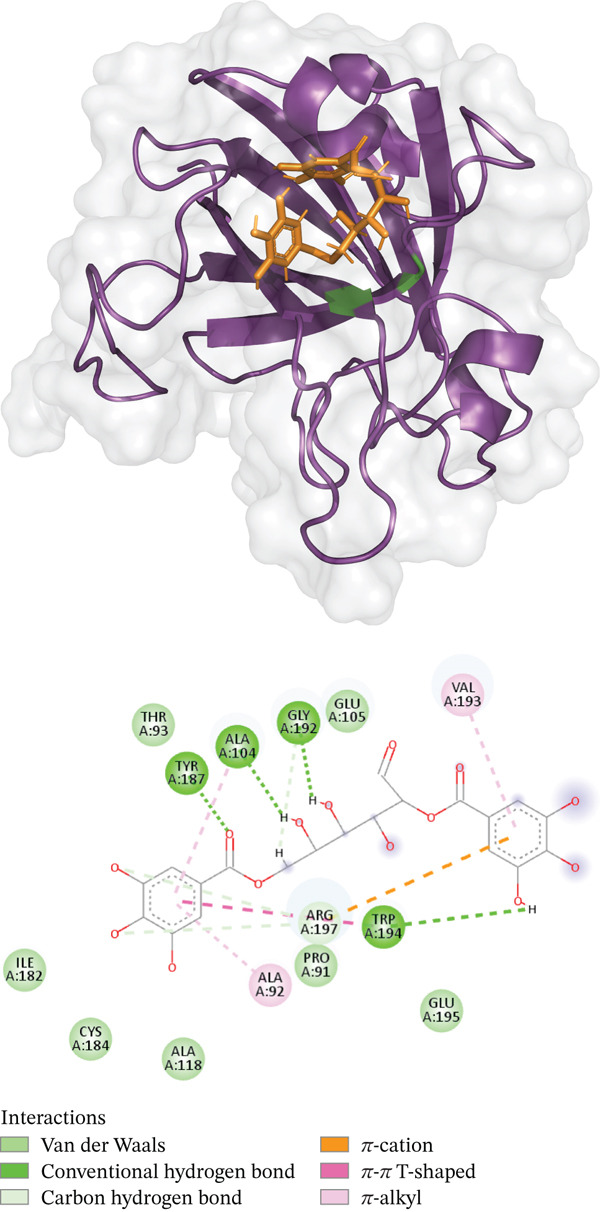
Docked conformation and interactions of the digalloyl‐glucose with sortase A from *Staphylococcus aureus* (chain A; PDB ID: 1T2P). The protein (purple) has the molecular surface displayed in the background. The ligand is orange, and the key catalytic residues His120 and Cys184 are green.

According to Figure 9, digalloyl‐glucose is positioned within a well‐defined cavity corresponding to the active site of SrtA. The ligand is in proximity to key catalytic residues, including His120 and Cys184, supporting the assignment of this cavity as the functional binding site and indicating potential interaction with the catalytic region of the enzyme. The residues predicted to interact with 2,6‐digalloyl‐D‐glucose within the active site of the enzyme were Pro91, Ala92, Thr93, Ala104, Glu105, Ala118, Ile182, Cys184, Tyr187, Gly192, Val193, Trp194, Glu195, and Arg197.

## 4. Discussion

The study of natural products with antibacterial activity has become increasingly relevant, given the rising resistance to conventional therapies. In this context, it is important to include extracts from *A. occidentale*, given their diverse biological activities and use in traditional medicine, with potential clinical applications [14–19, 31, 32].

The present study evaluated the antimicrobial and antivirulence potential of the EHAo against standard and clinically significant ESKAPE bacteria, including MRSA. Previous studies have described the antimicrobial properties of extracts from flowers and other aerial parts of *A. occidentale* [14–19, 32]. However, studies examining flower extracts—particularly against clinical isolates and virulence‐related phenotypes relevant to clinical results—remain scarce.

Chemical analysis of EHAo revealed 14 compounds, among them shikimic acid, gallic acid, quercetin, isoquercetin, and galloyl glucose derivatives. HPLC‐MS enabled effective detection of polyphenolic and other secondary metabolites. Extraction conditions and analytical techniques determine which compounds and at what concentrations are detected. Gallic acid and shikimic acid have been previously reported in *A. occidentale* flower extracts [14, 19], whereas glycosylated quercetin and isoquercetin have not. The actual chemical profiles, compared with prior studies, including the absence of anacardic acids [19], may reflect differences in extraction or analytical methods, or ecological variation in secondary metabolites driven by seasonal climatic changes [33].

The extract efficiently inhibited the growth of gram‐positive bacteria (*E. faecalis* and *S. aureus*) and, to a lesser extent, gram‐negative bacteria (*E. coli*), but had limited efficacy against *K. pneumoniae* in vitro. This result indicates that EHAo was more effective against Gram‐positive bacteria, including some clinical MRSA strains.

The EHAo′s selectivity for gram‐positive bacteria merits attention, especially given the high resistance of those microorganisms to available conventional antimicrobials [4, 5, 11, 12]. In addition, the extract′s effective activity against MRSA clinical isolates is also promising, given the challenges presented by antibiotic resistance. The observed EHAo selectivity may be due to polyphenolic compounds, which can interact differently with bacterial cell envelopes. In gram‐negative bacteria, the outer membrane restricts penetration of these substances, acting as a barrier much like it does for many antibiotics [34, 35].

Biofilm‐associated infections involve *E. coli*, *S. aureus*, and MRSA in approximately 100%, 90%, and 60.8% of cases, respectively [36]. This evidence motivated us to investigate the effects of EHAo on these clinically important bacteria, particularly given that more than 150 million people worldwide are affected each year by catheter‐associated urinary tract infections—the most common biofilm‐related disease linked to medical devices [37, 38].

In vitro, the extract inhibited adhesion and biofilm formation at all tested concentrations against standard *S. aureus, E. coli,* and clinical *MRSA strains.* Biomass and viability reductions show EHAo′s bactericidal activity, confirmed by the MIC/CBM ratio as reported by Sidiqui et al. [22]. Adhesion is essential for infection, and biofilms increase bacterial persistence and resistance to host defenses and antibiotics, and they both are considered important virulence factors. Thus, EHAo likely combines antivirulence and bactericidal activity [3]. Higher concentrations reduced adhesion similarly to conventional antibiotics, underscoring EHAo′s potential to prevent device‐associated infections, such as catheter‐related infections [4–7].

The antimicrobial activity of EHAo and its inhibitory effects on adhesion and biofilm formation in gram‐positive bacteria may be related to polyphenolic compounds, such as gallic acid and quercetin, and their derivatives. These substances can cause cellular dysfunction by altering bacterial membranes, increasing membrane permeability, and leading to bacterial death [39–41]. Polyphenolic compounds may also increase bacterial susceptibility to antibiotics [40, 42] and disrupt quorum‐sensing pathways that control biofilm formation and maturation [43]. This disruption helps explain the inhibition of some virulence factors.

*In vivo* assays used *S. aureus,* given its high prevalence in various infections [44, 45]. *S. aureus* pathogenicity relies on factors such as surface proteins, toxins, and enzymes that promote adhesion, tissue damage, and immune evasion [36, 44, 45].

The treatment with EHAo increased *T. molitor* survival after a lethal infection with *S. aureus* in a dose‐dependent manner. *T*. *molitor* is a convenient, rapid, and ethical model for initial *in vivo* screening [26]. The study with this larva can generate data relevant to subsequent vertebrate studies, despite differences in immune response, physiology, and metabolism compared with those of mammals. Therefore, validation in vertebrate models is still necessary to accurately assess therapeutic potential and translational relevance.

CFU quantification after *T. molitor* infection showed an exponential increase in bacterial growth at 6 h postinfection. This pattern is consistent with clinical observations, where patients with severe bacteremia—particularly those who are immunosuppressed or using medical devices—often progress to death within 24–72 h [46].

Although *T. molitor* is widely used as an infection model in studies of natural products [19, 27], we did not find any publications reporting the antibiotic potential of EHAo against lethal *S. aureus* infection in these larvae. The in vivo findings are relevant, given that *S. aureus* is associated with a variety of local and systemic infections. It also reaffirms the importance of *A. occidentale* as a rich source of bioactive compounds. These compounds may be used alone in new herbal medicines or as therapeutic adjuvants [14, 19, 47, 48].

The EHAo molecular effects were investigated in silico using SrtA as a target. SrtA is important for controlling *S. aureus* infection [10]. This enzyme anchors surface proteins needed for adhesion and biofilm formation in *S. aureus* and other Gram‐positive bacteria. Inhibition of SrtA prevents *S. aureus* from properly displaying surface proteins, highlighting its essential role in virulence [49].

All polyphenolic compounds in EHAo showed predicted affinity for the SrtA active site, especially digalloyl glucose, isoquercetin, and quercetin. These compounds may have many hydroxyl groups and aromatic rings, which enhance binding affinity and interactions with the active site. Thus, polyphenolic compounds—especially those with greater structural complexity—may inhibit SrtA and affect bacterial adhesion and biofilm formation [50].

Digalloyl‐glucose and quercetin exhibit membrane‐permeabilizing properties. They alter bacterial cell wall permeability and induce metabolic dysfunction [51–53], consequently increasing their antibacterial effects. The chemical composition may help to explain the observed effects in vivo during *S. aureus* infection. It is well known that polyphenols can inhibit SrtA activity [10] and the expression of *quorum-sensing* molecules, reducing the biofilm density and impairing the biofilm maturation [39, 41, 54]. Inhibition of *quorum-sensing* and biofilm formation is relevant for therapy, as it can reduce bacterial persistence and resistance. Based on those results it is reasonable to propose that EHAo′s compounds can be important resources for new compounds to be used alone or that could serve as adjuvants to conventional therapy.

Gallic and shikimic acids, as well as other compounds in the extract, are widely studied for their biological activities [39, 41, 42, 55]. Gallic acid disrupts bacterial membranes, whereas shikimic acid serves as an adjuvant for beta‐lactams against multidrug‐resistant bacteria, including MRSA [42, 55–57].

Polyphenols are known to inhibit. Although docking analyses yield predictive information, findings show that all compounds in EHAo bind to the active site of SrtA. Polyphenolic compounds also exhibit antioxidant, antimicrobial, and anti‐inflammatory properties. These effects are important for controlling inflammation caused by infection. Polyphenols act as natural defense mechanisms for plants. The EHAo may allow several compounds to act synergistically [55].

Based on these results, it is reasonable to propose that the protective effect of this extract is due to several compounds forming a phytocomplex [58]. Together, they can directly affect bacterial activity and interfere with virulence mechanisms, such as adhesion and biofilm formation. However, further studies in vertebrate systems are needed to understand pharmacological properties, host immune responses, and therapeutic potential.

Altogether, the results show that EHAo has antimicrobial and antivirulence activities, particularly against gram‐positive bacteria, including *S. aureus* and MRSA. The combined effects on bacterial virulence factors and growth, together with its low in vivo toxicity and the prediction of its inhibitory action on SrtA, support the idea that EHAo is a promising source of bioactive compounds.

## 5. Conclusions

The ethanolic extract of *A. occidentale* flowers was more effective against Gram‐positive bacteria, including *S. aureus*, MRSA, *E. faecalis*, and the gram‐negative *E. coli*. The extract also effectively inhibited virulence factors of those bacteria, including adhesion and biofilm formation. An important activity against *S. aureus* was detected, as the treatment improved the survival of lethally infected *T. molitor* larvae. In addition, the in silico prediction showed that all compounds present in EHAo can potentially inhibit the active site of SrtA, which is common in other gram‐positive bacteria, suggesting therapeutic potential as a target. The results reinforce the potential of natural products as complementary strategies for developing new antimicrobial approaches, particularly those effective in inhibiting bacterial virulence rather than viability.

**Nomenclature**
CFU: colony forming units
CIPRO: ciprofloxacin
EHAo: ethanolic extract of *Anacardium occidentale* flowers
MH: Mueller–Hinton
MRSA: methicillin‐resistant *Staphylococcus aureus*
UFMA: Universidade Federal do Maranhão

## Author Contributions

Conceptualization: Rosane Nassar Meireles Guerra and Pâmela Gomes Santos; methodology: Pâmela Gomes Santos, Josivan Regis Farias, Simone Batista Muniz, Danielle Cristine Gomes Franco, Ariadina Jansen Campos Fontes, Liane Maria Rodrigues Santos, and Alexandra Martins dos Santos Soares; validation: Flavia Raquel Fernandes Nascimento and Rosane Nassar Meireles Guerra; chemical analysis: Claudia Quintino Rocha and Pâmela Gomes Santos; molecular docking and in silico analysis: Alexandra Martins dos Santos Soares; formal analysis: Rosane Nassar Meireles Guerra, Pâmela Gomes Santos; resources and funding acquisition: Rosane Nassar Meireles Guerra, Flavia Raquel Fernandes Nascimento, and Claudia Quintino Rocha; data curation: Rosane Nassar Meireles Guerra and Pâmela Gomes Santos; writing—review and editing: Rosane Nassar Meireles Guerra, Pâmela Gomes Santos; project administration: Rosane Nassar Meireles Guerra.

## Funding

This study was supported by the FAPEMA (Fundação de Amparo à Pesquisa do Maranhão) Project NATUMED (Process No. IECT‐02885/17), CNPq (Conselho Nacional de Desenvolvimento e Tecnológico) FitoAmazônia Research Network (CNPq Process 445615/2024‐9), Universidade Federal do Maranhão, and CAPES (Coordenação de Aperfeiçoamento de Pessoal de Nível Superior) (Financial Code 001).

## Disclosure

All authors have read and agreed to the published version of the manuscript.

## Ethics Statement

The study is registered with the National System for the Management of Genetic Heritage and Associated Traditional Knowledge (SisGen) (Registration No. AFE7A08) as regulated by Brazilian Law No. 13.123/15.

## Consent

The authors have nothing to report.

## Conflicts of Interest

The authors declare no conflicts of interest.

## Data Availability

The data that support the findings of this study are available from the corresponding author upon reasonable request.

## References

[1] Oliveira D. M. P. , Forde B. M. , Kidd T. J. , Harris P. N. A. , Schembri M. A. , Beatson S. B. , Paterson D. L. , and Walker M. J. , Antimicrobial Resistance in ESKAPE Pathogens, Clinical Microbiology Reviews. (2020) 33, no. 3, 10–1128, 10.1128/CMR.00181-19. PMC7227449 32404435

[2] Karnwal A. , Kumar G. , Pant G. , Hossain K. , Ahmad A. , and Alshammari M. B. , Perspectives on Usage of Functional Nanomaterials in Antimicrobial Therapy for Antibiotic-Resistant Bacterial Infections, ACS Omega. (2023) 8, no. 15, 13492–13508, 10.1021/acsomega.3c00110. 37091369 PMC10116640

[3] Miller W. R. and Arias C. A. , ESKAPE Pathogens: Antimicrobial Resistance, Epidemiology, Clinical Impact and Therapeutics, Nature Reviews Microbiology. (2024) 22, no. 10, 598–616, 10.1038/s41579-024-01054-w, 38831030. 38831030 PMC13147291

[4] Sousa M. , Nascimento G. , Bim F. , Oliveira L. , and Oliveira A. , Hospital Infections Related to Invasive Procedures in Intensive Care Units: A Literature Review, Journal of Infection Prevention and Health. (2017) 3, 49–58.

[5] Pinheiro L. , Martins C. , Martins C. , Caires P. , Aragão V. , Aragão O. , Ferraz R. , Felisberto Y. , Neto F. , and Chaves M. , Factors and Risk and Mortality in Critically Ill Patients With Infections by Multidrug-Resistant Microorganisms, Electronic Journal Collection Health. (2021) 13, no. 4, e7319, 10.25248/reas.e7319.2021.

[6] Ribeiro E. A. , Torres G. C. , Rodrigues G. F. , and Alves J. A. G. , Secondary Infections Caused by ESKAPE Group Bacteria and Impact on the Health of Patients With COVID-19 Complications, Research, Society and Development. (2022) 11, no. 15, e474111537997, 10.33448/rsd-v11i15.37997.

[7] Li G. , Lai Z. , and Shan A. , Advances of Antimicrobial Peptide-Based Biomaterials for the Treatment of Bacterial Infections, Advanced Science. (2023) 10, no. 11, e2206602, 10.1002/advs.202206602. 36722732 PMC10104676

[8] Bereanu A. S. , Bereanu R. , Mohor C. , Vintilă B. I. , Codru I. R. , Olteanu C. , and Sava M. , Prevalence of Infections and Antimicrobial Resistance of ESKAPE Group Bacteria Isolated From Patients Admitted to the Intensive Care Unit of a County Emergency Hospital in Romania, Antibiotics. (2024) 13, no. 5, 400–420, 10.3390/antibiotics13050400, 38786129. 38786129 PMC11117271

[9] Nazir A. , Song J. , Chen Y. , and Liu Y. , Phage-Derived Depolymerase: Its Possible Role for Secondary Bacterial Infections in COVID-19 Patients, Microorganisms. (2023) 11, no. 2, 1–13, 10.3390/microorganisms11020424. PMC9961776 36838389

[10] Chan A. H. , Wereszczynski J. , Amer B. R. , Yi S. W. , Jung M. E. , McCammon J. A. , and Clubb R. T. , Discovery of Staphylococcus aureus Sortase A Inhibitors Using Virtual Screening and the Relaxed Complex Scheme, Chemical Biology & Drug Design. (2013) 82, no. 4, 418–428, 10.1111/cbdd.12167, 23701677. 23701677 PMC3841297

[11] Hutchings M. I. , Truman A. W. , and Wilkinson B. , Antibiotics: Past, Present and Future, Current Opinion in Microbiology. (2019) 51, 72–80, 10.1016/j.mib.2019.10.008. 31733401

[12] Agência Nacional de Vigilância Sanitária , National Guideline for the Elaboration of an Antimicrobial Management Program in Health Services, 2023, ANVISA.

[13] Dong F. R. , Gao L. , Wang L. , Jiang Y. Y. , and Jin Y. S. , Natural Products as Antifungal Agents Against Invasive Fungi, Current Topics in Medicinal Chemistry. (2023) 23, no. 19, 1859–1917, 10.2174/1568026623666230417105227. 37070444

[14] Silva R. A. , Liberio S. , Amaral F. M. M. , Nascimento F. , Torres L. , Monteiro-Neto V. , and Guerra R. N. M. , Antimicrobial and Antioxidant Activity of Anacardium occidentale L. Flowers in Comparison Bark and Leaves Extracts, Journal of Biosciences and Medicines. (2016) 4, no. 4, 87–99, 10.4236/jbm.2016.44012.

[15] Freire J. C. P. , Junior J. K. O. , Santiago C. P. L. , Freire S. C. P. , and Lima E. O. , Ethnobotanical Study of the Cashew Tree (Anacardium occidentale L.): A Natural Tree From Brazil, Revista UNINGÁ Review. (2017) 29, no. 3, 123–126.

[16] Araújo S. , Sousa I. , Gonçalves R. , France A. , Negreiros P. , and Brito A. , Pharmacological and Technological Applications of Cashew Gum (Anacardium occidentale), Revista GEINTEC. (2018) 8, no. 1, 4292–4305, 10.7198/geintec.v8i1.1121.

[17] Araújo J. S. C. , Castilho A. R. F. , Lira A. B. , Pereira A. V. , Azevedo T. K. B. , and Costa E. M. M. B. , Antibacterial Activity Against Cariogenic Bacterium and Cytotoxic and Genotoxic Potential of Anacardium occidentale L. and Anadenanthera macrocarpa, Archives of Oral Biology. (2017) 85, 113–119, 10.1016/j.archoralbio.2017.09.030. 29054025

[18] Ribeiro A. D. , Junior E. C. F. , Junior J. G. R. , Costa B. P. , Freire J. C. P. , Melo W. O. S. , and Pereira J. V. , Potencial Antimicrobiano do Anacardium Occidentale Lin. Contra Patógenos Orais, Research, Society and Development. (2020) 9, no. 8, e684986459, 10.33448/rsd-v9i8.6459.

[19] Oliveira A. S. , Nascimento J. R. , Trovão L. O. , Alves P. C. S. , Maciel M. C. G. , Silva L. D. M. , Marques A. A. , Santos A. P. S. A. , Silva L. A. , Nascimento F. R. F. , and Guerra R. N. M. , The Anti-Inflammatory Activity of Anacardium Occidentale L. Increases the Lifespan of Diabetic Mice with Lethal Sepsis, Journal of Ethnopharmacology. (2019) 236, 345–353, 10.1016/j.jep.2019.03.014. 30872173

[20] Clinical and Laboratory Standards Institute , Performance Standards for Antimicrobial Susceptibility Testing. Twenty-Second Informational Supplement Clinical and Laboratory Standards Institute, 2015, Clinical and Laboratory Standards Institute.

[21] Monteiro-Neto V. , de Sousa C. D. , Gonzaga L. F. , Silveira B. C. , Souza N. C. F. , Pontes J. P. , Santos D. M. , Martins W. C. , Pessoa J. F. V. , Junior A. R. C. , Almeida V. S. S. , Oliveira N. M. T. , Araújo T. S. , Maria-Ferreira D. , Mendes S. J. F. , Ferro T. A. F. , and Fernandes E. S. , Cuminaldehyde Potentiates the Antimicrobial Actions of Ciprofloxacin Against Staphylococcus aureus and Escherichia coli, PloS One. (2020) 15, no. 5, e0232987, 10.1371/journal.pone.0232987, 32407399. 32407399 PMC7224478

[22] Siddiqui Z. N. , Farooq F. , Musthafa T. N. M. , Ahmad A. , and Khan A. U. , Synthesis, Characterization and Antimicrobial Evaluation of Novel Halopyrazole Derivatives, Journal of Saudi Chemical Society. (2013) 17, no. 2, 237–243, 10.1016/j.jscs.2011.03.016.

[23] Xavier A. L. , Silva N. C. O. , Soares R. R. , Abreu S. , Araújo E. A. , Júnior J. B. O. , and Siqueira A. B. S. , Atividade Antibacteriana do Extrato Etanólico da Própolis Vermelha Brasileira Contra Bactérias Produtoras de *β*-Lactamase e Carbapenemase de Espectro Estendido Multidroga-Resistentes, Scientia Plena. (2023) 19, no. 4, 044501, 10.14808/sci.plena.2023.044501.

[24] Silva T. F. and Filho J. R. N. C. , Products Derived From Buchenavia tetraphylla Leaves Have In Vitro Antioxidant Activity and Protect Tenebrio molitor Larvae Against Escherichia coli-Induced Injury, Pharmaceuticals. (2020) 13, no. 3, 10.3390/ph13030046. PMC7151707 32188166

[25] Brai A. , Poggialini F. , Vagaggini C. , Pasqualini C. , Simoni S. , Francardi V. , and Dreassi E. , Tenebrio Molitor as a Simple and Cheap Preclinical Pharmacokinetic and Toxicity Model, International Journal of Molecular Sciences. (2023) 24, no. 3, 10.3390/ijms24032296. PMC9917132 36768618

[26] Souza P. C. , Morey A. T. , Castanheira G. M. , Bocate K. P. , Panagio L. A. , Ito F. A. , Furlanetto M. C. , Yamada-Gata S. F. , Costa I. N. , Mora-Montes H. M. , and Almeida R. S. , Tenebrio molitor (Coleoptera: Tenebrionidae) as an Alternative Host to Study Fungal Infections, Journal of Microbiological Methods. (2015) 118, 182–186, 10.1016/j.mimet.2015.10.004, 26453946. 26453946

[27] McGonigle J. E. , Purves J. , and Rolff J. , Intracellular Survival of Staphylococcus aureus During Persistent Infection in the Insect Tenebrio molitor, Developmental & Comparative Immunology. (2016) 59, 34–40, 10.1016/j.dci.2016.01.002. 26778297

[28] Bitencourt-Ferreira G. and Azevedo W. F. , Molegro Virtual Docker for Docking, Docking Screens for Drug Discovery2019, Springer, 10.1007/978-1-4939-9752-7_10.

[29] Hanwell M. D. , Curtis D. E. , Lonie D. C. , Vandermeersch T. , Zurek E. , and Hutchison G. R. , Avogadro: An Advanced Semantic Chemical Editor, Visualization, and Analysis Platform, Journal of Cheminformatics. (2012) 4, no. 1, 10.1186/1758-2946-4-17, 22889332. PMC3542060 22889332

[30] SAS Institute Inc , SAS, User′s Guide: Statistics. Version 9.0, 2002, SAS Institute.

[31] Araujo J. M. , Silva A. P. , Cândido M. B. , Silva T. W. M. , and Junior F. P. A. , Ethnobotanical Study of Anacardium occidentale, Research, Society and Development. (2020) 9, no. 8, e374985802, 10.33448/rsd-v9i8.5802.

[32] Pereira A. V. , Azevedo T. K. B. , Higino S. S. , Santana G. M. , Trevisa L. F. A. , Azevedo S. S. , Pereira M. V. , and Paula A. F. R. , Tannins From Cashew Bark: Antimicrobial Activity, Journal of Agroindustry and Technology. (2015) 36, no. 1, 121–127.

[33] Dong P. , Wang L. , Qiu D. , Liang W. , Cheng J. , Wang H. , Guo F. , and Chen Y. , Evaluation of the Environmental Factors Influencing the Quality of Astragalus membranaceus var. mongholicus Based on HPLC and the Maxent Model, BMC Plant Biology. (2024) 24, no. 1, 10.1186/s12870-024-05355-3, 39044138. PMC11264576 39044138

[34] Makarewicz M. , Drożdż I. , Tarko T. , and Duda-Chodak A. , The Interactions Between Polyphenols and Microorganisms, Especially Gut Microbiota, Antioxidants. (2021) 10, no. 2, 188–258, 10.3390/antiox10020188. 33525629 PMC7911950

[35] Li K. , Xing S. , Wang M. , Peng Y. , Dong Y. , and Li X. , Anticomplement and Antimicrobial Activities of Flavonoids from Entada Phaseoloides, Natural Products Communications. (2012) 7, 867–871. 22908567

[36] Cangui-Pachi S. P. , Ñacato-Toapanta A. L. , Enríquez-Martinez L. J. , Reyes J. , Garzon-Chavez D. , and Machado A. , Biofilm-Forming Microorganisms Causing Hospital-Acquired Infections From Intravenous Catheter: A Systematic Review, Current Research in Microbial Sciences. (2022) 3, 100171, 10.1016/j.crmicr.2022.100171. 36518176 PMC9743049

[37] Jamal M. , Ahmad W. , Andleeb S. , Jalil F. , Imran M. , Nawaz M. A. , Hussain T. , Ali M. , Rafiq M. , and Kamil M. A. , Bacterial Biofilm and Associated Infections, Journal of the Chinese Medical Association. (2018) 81, no. 1, 7–11, 10.1016/j.jcma.2017.07.012. 29042186

[38] Mishra A. , Aggarwal A. , and Khan F. , Medical Device-Associated Infections Caused by Biofilm-Forming Microbial Pathogens and Controlling Strategies, Antibiotics. (2024) 139, no. 7, 1–16, 10.3390/antibiotics13070623. PMC11274200 39061305

[39] Roy R. , Tiwari M. , Donelli G. , and Tiwari V. , Strategies for Combating Bacterial Biofilms: A Focus on Anti-Biofilm Agents and Their Mechanisms of Action, Virulence. (2018) 9, no. 1, 522–554, 10.1080/21505594.2017.1313372, 28362216. 28362216 PMC5955472

[40] Movahedi A. , Almasi Zadeh Yaghuti A. , Wei H. , Rutland P. , Sun W. , Mousavi M. , Li D. , and Zhuge Q. , Plant Secondary Metabolites With an Overview of Populus, International Journal of Molecular Sciences. (2021) 22, no. 13, 10.3390/ijms22136890. PMC8268465 34206964

[41] Tavares T. D. , Antunes J. C. , Padrão J. , Ribeiro A. I. , Zille A. , Amorim M. T. P. , Ferreira F. , and Felgueiras H. P. , Activity of Specialized Biomolecules Against Gram-Positive and Gram-Negative Bacteria, Antibiotics. (2020) 9, no. 6, 314–316, 10.3390/antibiotics9060314. 32526972 PMC7344598

[42] Hou L. , Ye M. , Wang X. , Zhu Y. , Sun X. , Gu R. , Chen L. , and Fang B. , Synergism With Shikimic Acid Restores *β*-Lactam Antibiotic Activity Against Methicillin-Resistant Staphylococcus aureus, Molecules. (2024) 29, no. 7, 10.3390/molecules29071528. PMC11013880 38611807

[43] Lima E. M. F. , Winans S. C. , and Pinto U. M. , Quorum Sensing Interference by Phenolic Compounds - a Matter of Bacterial Misunderstanding, Heliyon. (2023) 9, no. 7, 17657–17672, 10.1016/j.heliyon.2023.e17657, 37449109. PMC10336516 37449109

[44] Bashabsheh R. H. F. , Al-Fawares O. , Natsheh I. , Bdeir R. , Al-Khreshieh R. O. , and Bashabsheh H. H. F. , Staphylococcus aureus Epidemiology, Pathophysiology, Clinical Manifestations and Application of Nano-Therapeutics as a Promising Approach to Combat Methicillin Resistant Staphylococcus aureus, Pathogens and Global Health. (2024) 118, no. 3, 209–231, 10.1080/20477724.2023.2285187, 38006316. 38006316 PMC11221481

[45] Yang W. Y. , Kim C. K. , Ahn C. H. , Kim H. , Shin J. , and Oh K. B. , Flavonoid Glycosides Inhibit Sortase A and Sortase A-Mediated Aggregation of Streptococcus mutans, an Oral Bacterium Responsible for Human Dental Caries, Journal of Microbiology and Biotechnology. (2016) 26, no. 9, 1566–1569, 10.4014/jmb.1605.05005, 27291675. 27291675

[46] Oliveira R. D. , Bustamante P. F. O. , and Besen B. A. M. P. , Tackling Healthcare-Associated Infections in Brazilian Intensive Care Units: We Need More Than Collaboration, Revista Brasileira de Terapia Intensiva. (2012) 34, no. 3, 313–315, 10.5935/0103-507X.20220235-en. PMC9749086 36351063

[47] Furtado M. A. M. , Alves F. C. S. , Martins J. L. , Vaconcelos M. A. , Ramos V. S. C. , Sousa G. S. , Silva A. L. C. , Farias W. R. L. , Teixeira E. H. , Cavada B. S. , and Santos R. P. , Effect of Cashew (Anacardium occidentale L.) Peduncle Bagasse Extract on Streptococcus mutans and Its Biofilm. Braz, Brazilian Journal of Biosciences. (2014) 2, no. 1, 9–13.

[48] Borges I. G. , Cavalcante J. T. , Araújo T. C. , and Sousa F. O. , Bactericidal and Antibiofilm Activity of Anacardic Acid–Loaded Seín Nanoparticles Against Enterococcus faecalis Ex Vivo, Journal of Computational and Theoretical Nanoscience. (2020) 17, no. 7, 2918–2925, 10.1166/jctn.2020.9270.

[49] Schneewind O. and Missiakas D. , Sortases, Surface Proteins, and Their Roles in Staphylococcus aureus Disease and Vaccine Development, Microbiology Spectrum. (2019) 7, no. 1, 1–13, 10.1128/microbiolspec.PSIB-0004-2018. PMC6386163 30737913

[50] Nitulescu G. , Nicorescu I. M. , Olaru O. T. , Ungurianu A. , Mihai D. P. , Zanfirescu A. , Nitulescu G. M. , and Margina D. , Molecular Docking and Screening Studies of New Natural Sortase A Inhibitors, International Journal of Molecular Sciences. (2017) 18, no. 10, 2217–2231, 10.3390/ijms18102217, 29065551. 29065551 PMC5666896

[51] Kováč J. , Slobodníková L. , Trajčíková E. , Rendeková K. , Mučaji P. , Sychrová A. , and Fialová S. B. , Therapeutic Potential of Flavonoids and Tannins in Management of Oral Infectious Diseases—A Review, Molecules. (2022) 28, no. 158, 1–21, 10.3390/molecules28010158. 36615352 PMC9821998

[52] Hooda H. , Singh P. , and Bajpai S. , Effect of Quercetin Impregnated Silver Nanoparticle on Growth of Some Clinical Pathogens, Materials Today: Proceedings. (2020) 31, 625–630, 10.1016/j.matpr.2020.01.551.

[53] Nguyen T. L. A. and Bhattacharya D. , Antimicrobial Activity of Quercetin: An Approach to Its Mechanistic Principle, Molecules. (2022) 27, no. 8, 10.3390/molecules27082494. PMC9029217 35458691

[54] Lal A. , Singh S. , Franco F. C. , and Bhatia S. , Potential of Polyphenols in Curbing Quorum Sensing and Biofilm Formation in Gram-Negative pathogens, Asian Pacific Journal of Tropical Biomedicine. (2021) 11, no. 6, 231–243, 10.4103/2221-1691.314044.

[55] Sang H. , Jin H. , Song P. , Xu W. , and Wang F. , Gallic Acid Exerts Antibiofilm Activity by Inhibiting Methicillin-Resistant Staphylococcus aureus Adhesion, Scientific Reports. (2024) 14, no. 1, 1–11, 10.1038/s41598-024-68279-w. 39060363 PMC11282228

[56] Hadidi M. , Liñán-Atero R. , Tarahi M. , Christodoulou M. C. , and Aghababaei F. , The Potential Health Benefits of Gallic Acid: Therapeutic Insights and Food Applications, Antioxidants. (2024) 13, no. 8, 10.3390/antiox13081001. PMC11352096 39199245

[57] Stiller A. , Garrison K. , Gurdyumov K. , Kenner J. , Yasmin F. , Yates P. , and Song B. H. , From Fighting Critters to Saving Lives: Polyphenols in Plant Defense and Human Health, International Journal of Molecular Sciences. (2021) 22, no. 16, 10.3390/ijms22168995, 34445697. PMC8396434 34445697

[58] Motta E. P. , Farias J. R. , Costa A. A. C. , Silva A. F. , Oliveira Lopes A. J. , Cartágenes M. S. S. , Nicolete R. , Abreu A. G. , Fernandes E. S. , Nascimento F. R. F. , Rocha C. Q. , Monteiro C. A. , and Guerra R. N. M. , The Anti-Virulence Effect of Vismia guianensis Against Candida albicans and Candida glabrata, Antibiotics. (2022) 11, no. 12, 1834–1847, 10.3390/antibiotics11121834, 36551490. 36551490 PMC9774440

